# Functionalized multi-walled carbon nanotubes and hydroxyapatite nanorods reinforced with polypropylene for biomedical application

**DOI:** 10.1038/s41598-020-80767-3

**Published:** 2021-01-12

**Authors:** Fahad Saleem Ahmed Khan, N. M. Mubarak, Mohammad Khalid, Rashmi Walvekar, E. C. Abdullah, Awais Ahmad, Rama Rao Karri, Harshini Pakalapati

**Affiliations:** 1 Department of Chemical Engineering, Faculty of Engineering and Science, Curtin University, 98009 Miri Sarawak, Malaysia; 2 Graphene & Advanced 2D Materials Research Group (GAMRG), School of Engineering and Technology, Sunway University, No. 5, Jalan Universiti, Bandar Sunway, 47500 Petaling Jaya, Selangor Malaysia; 3 Department of Chemical Engineering, School of Energy and Chemical Engineering, Xiamen University Malaysia, Jalan Sunsuria, Bandar Sunsuria, 43900 Sepang, Selangor Malaysia; 4 Department of Chemical Process Engineering, Malaysia-Japan International Institute of Technology (MJIIT) Universiti Teknologi Malaysia (UTM), Jalan Sultan Yahya Petra, 54100 Kuala Lumpur, Malaysia; 5 Department of Chemistry, The University of Lahore, Lahore, 54590 Pakistan; 6 Petroleum, and Chemical Engineering, Faculty of Engineering, Universiti Teknologi Brunei, Bandar Seri Begawan, BE1410 Brunei Darussalam; 7 Department of Chemical and Environmental Engineering, Faculty of Engineering, University of Nottingham Malaysia Campus, Jalan Brogans, 43500 Semenyih, Selangor Malaysia

**Keywords:** Health care, Engineering, Materials science, Nanoscience and technology

## Abstract

Modified multi-walled carbon nanotubes (f-MWCNTs) and hydroxyapatite nanorods (n-HA) were reinforced into polypropylene (PP) with the support of a melt compounding approach. Varying composition of f-MWCNTs (0.1–0.3 wt.%) and nHA (15–20 wt.%) were reinforced into PP, to obtain biocomposites of different compositions. The morphology, thermal and mechanical characteristics of PP/n-HA/f-MWCNTs were observed. Tensile studies reflected that the addition of f-MWCNTs is advantageous in improving the tensile strength of PP/n-HA nanocomposites but decreases its Young’s modulus significantly. Based on the thermal study, the f-MWCNTs and n-HA were known to be adequate to enhance PP’s thermal and dimensional stability. Furthermore, MTT studies proved that PP/n-HA/f-MWCNTs are biocompatible. Consequently, f-MWCNTs and n-HA reinforced into PP may be a promising nanocomposite in orthopedics industry applications such as the human subchondral bone i.e. patella and cartilage and fabricating certain light-loaded implants.

## Introduction

Bone fractures are prevalent nowadays. In Europe, the occurrence of bone failures is stated to be 2.68 million per annum. Bone disease and trauma are the two main causes of bone fractures. Moreover, the osteoporosis prevalence is causing most of the fractures, and the statistics showed 7–19% and 23–35% for men and women, respectively, over the age of 50 years^1^. It is expected that by the year 2040, globally, there will be a significant elevation in the fracture threshold of over 300 million people^2^. Due to aging and life-span increasing, fragility is turning a blockade to mobility and quality of life, particularly for aged people.

Recently, Biomaterials usage for bone replacement has attained considerable attention. Biomaterials application as bone implant required to be biocompatible as well as adequate mechanical stability to support human body weight. Commonly used materials in the orthopedic industry include polymer composite and metal or alloy^3,4^. Due to extremely high mechanical stability and stiffness, metal/alloy material does not possess identical natural bone characteristics; therefore, it leads to implant loosening and/or bone density reduction^5^. In contrast, polymer composites offer a substitute solution as their mechanical features can be tailored through suitable fillers and polymers types. Polymer composites comprising hydroxyapatite fillers considered the primary polymer-based biomaterials for bone tissue applications. Bone tissue is comprising of collagen matrix and incorporation of hydroxyapatite nano-platelets. Commercialized hydroxyapatite-collagen composite is usually possess poor mechanical stability, thus is primarily applied as biodegradable scaffolds for bone tissue applications. Founder of HAPEX polymer, use 40% vol. hydroxyapatite microparticles and disperse in high-density poly-ethylene matrix^6^. The high amount of hydroxyapatite microparticles follows to seven times increase in HDPE elastic modulus. Thus, HAPEX polymer applications are limited in the orthopedic industry due to un-satisfied mechanical stability. Furthermore, Tang et al.^7^ described that high content of hydroxyapatite microparticles debond with ease and as a result, inadequate stress-transfer phenomenon within the matrix-filler boundary at tensile analyses. For polymer composites, a high amount of hydroxyapatite microparticles behave as stress concentrators and eagerly fracture during loading.

Nanotechnology advancement allows material scientists to produce nanohydroxyapatite with excellent biocompatibility of different morphologies^8^. Research studies has stated that synthetic nano-hydroxyapatite is active for securing osteoblasts and helping their expansion. Therefore, the incorporation of nano-hydroxyapatite with polymer composites are capable biomaterials for orthopedic applications. Recently, nano-hydroxyapatite have been reinforced in polyethylene, polyamide, and poly-hydroxy-ethyl methacrylate as they offer better bioactivity and mechanical strength^9,10^. Compared to the abovementioned polymer, polyamide (PA) holds an identical chemical structure as to collagen. Thus, high moisture adsorption of PA-based nanocomposites limited their clinical applications and is referred the only drawback. Polyethylene (PE) is comparatively soft and ductile, and hence high amount of nanofiller is required to elevate its tensile strength.

Another polymer named, polypropylene (PP), is a versatile polyolefin broadly used in construction, household goods, food packaging, and automotive applications due to its high dimensional stability, shape, and chemical resistance^11^. PP was initially founded by Giulio Natta in 1954^12^. However, its dominance over other polymer materials used in the biomedical industry was introduced in 1962 by Francis C. Usher^13^, partially due to its capability to be autoclaved. Moreover, PP is also considered for various biomedical applications such as bone cement, plastic surgery, surgical suture, and bone plates^14,15^. PP is a recommended option for pelvic floor repair owing to its use in inguinal hernia and abdominal wall repair. Compared to PE, PP displays improved fatigue resistance. Accordingly, it is more suitable than PE as the matrix material that is exposed to cyclic loading upon implantation into the human body^16^. Liu et al.^17^ demonstrated the mechanical strength of injection molded PP incorporate with 10–25 vol% hydroxyapatite (24.5 µm), and the results showed that Young’s modulus inclines but PP tensile strength declines with inclining filler content. Liu’s work reflected that the high content of hydroxyapatite microparticles are unproductive to reinforce PP. In another study conducted by Li et al.^11^ where PP composite produced with various hydroxyapatite nanorods (nHA) contents to examine the morphology, mechanical, and bioactivity characteristics. Tensile studies of Li’s work displayed that the addition of nHA reinforces and stiffens PP, however, tensile ductility reduces. Mostly, bioactivity, biocompatibility, and mechanical performance of nHA/polymer depend significantly on the volume fraction of nHA and kind of polymers.

Carbon nanotubes (CNTs) with high mechanical integrity, low density, high flexibility, and high surface area and aspect ratio have been broadly considered as incorporation fillers for polymers to produce nanocomposites with functional characteristics^18^. Carbon is the primary component of biomolecules; hence CNTs are known to be biocompatible with human tissues. CNTs offer wide-spectrum opportunities for orthopedic applications as they encourage adhesion and expansion of myoblasts, neurons, and osteoblasts^19^. Consequently, bone regeneration on CNT-based composites is progressively favored. Recently, a significant number of researchers have analyzed binary CNT/polymer composites fabricated for biomedical related applications. Based on diameter dimensions, CNTs are categorized as single-walled carbon nanotubes (SWCNTs) and multi-walled carbon nanotubes (MWCNTs). In comparison to SWCNTs, MWCNTs fabricated using chemical vapor deposit (CVD) have offered at economical price commercially.

CNTs, like any other material, face challenges regarding their biological compatibility. The CNTs interaction with host tissue, structure effect (MWCNTs vs SWCNTs), degree of aggregation, size, and effect of functional groups are a few of the considerations that will determine their compatibility for bone engineering. Un-modified (pristine) CNTs carry limitations like the capability to generate stable bundles or aggregates due to extremely strong interactions, i.e. strong π- π stacking and Van-der Waals force. The consequences of aggregation brought undesirable changes; it affects the aspect ratio and decreases the properties of the nanocomposite. In the past, several techniques had discovered, among them, the well-known and widely applied are covalent and non-covalent functionalization. All these approaches are with organic as well as inorganic compounds that help to achieve enhanced solubility and dispersibility^20^. For instance, un-modified CNTs used in living organisms and cells bring noxious effects; therefore, functionalization is to be concerned. Besides, the covalent functionalization approach is more recommended due to the availability of different epoxy, hydroxyl, and carboxylic groups. Studies conducted by Singh et al. concluded that functionalized CNTs can be employed in biodegradable scaffolds, and can achieve clearance via their dispersion in water and ultimate removal from the body^21^.

As aforementioned the characteristics of PP, it has been demonstrated that when implanted in the human body, PP holds its tensile property over a long period, i.e. more than 2 months^22,23^. This study goals to examine the n-HA and f-MWCNTs addition as filler on PP’s mechanical, thermal, and biocompatibility characteristics. Therefore, different compositions of f-MWCNTs (i.e. 0.1–0.3 wt.%) and n-HA (15–20 wt.%) were put in PP together to form biocomposites using a melt processing approach. In comparison to other existing approaches such as solution mixing, in-situ polymerization, latex technology, and melt compounding (also known as melt mixing) are the most recommended and versatile method employed to develop nanocomposites. The method uses high shear force and temperature to disperse materials in the polymer matrix. Wide applicability, no solvent required, widest selection of materials, and good dispersion are the main advantages of this method^24,25^.

By merging f-MWCNTs and n-HA properties in PP to form nanocomposites, various features like dimensional strength, mechanical property, and biocompatibility can be examined. In the past, several nanocomposites have been developed (for instance, polymethyl methacrylate (PMMA)/n-HA/MWCNTs) and the results concluded that the nanocomposites were not suitable for orthopedics applications to stand load-bearing implants^20,26^.

To the best of our knowledge, there has been no research study towards putting the PP, nHA, and f-MWCNTs to fabricate PP/n-HA/f-MWCNTs nanocomposites. Also, there is no reported study in open literature comparing the morphology, mechanical and thermal properties of PP/n-HA/f-MWCNTs nanocomposites. This article also presents the hybridizing additions of n-HA and f-MWCNTs as fillers on PP’s mechanical, thermal, and biocompatibility characteristics.

## Results and discussion

### X-ray diffraction (XRD)

**Figure 1:**
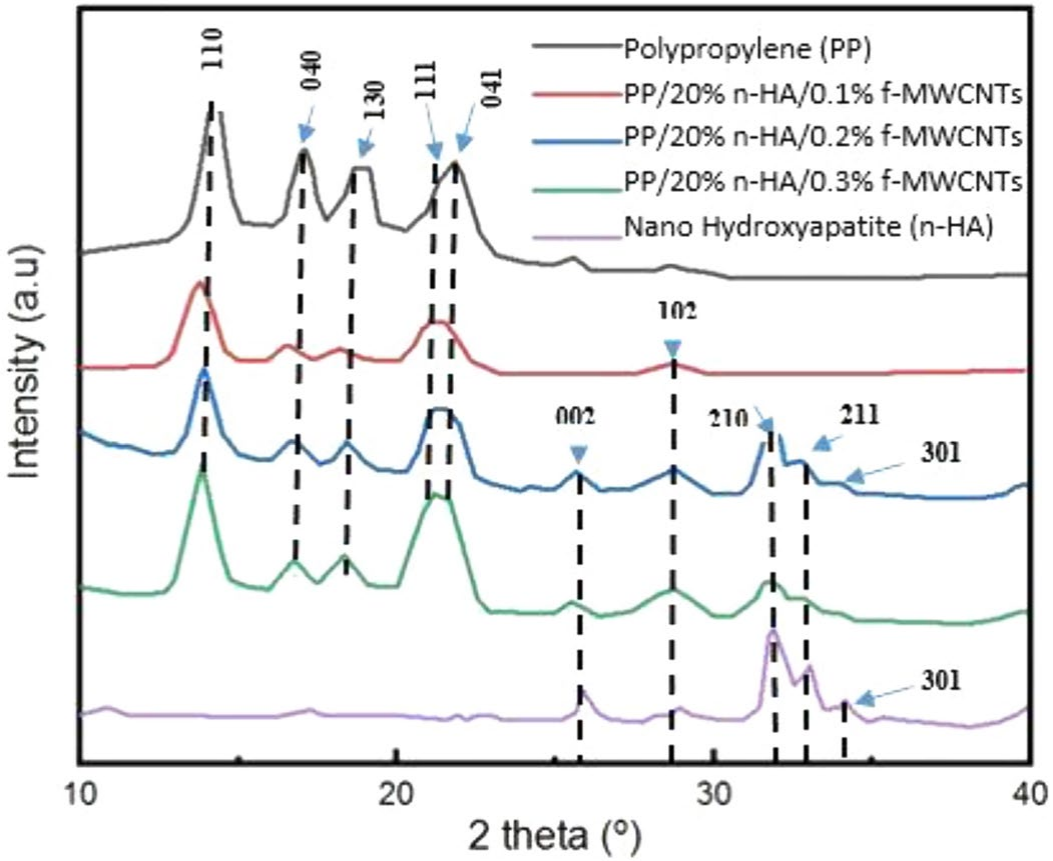
X-ray Pattern for Polypropylene (PP), PP/20% n-HA/0.1% f-MWCNTs, PP/20% n-HA/0.2% f-MWNTs, PP/20% n-HA/0.3% f-MWCNTs and Nano Hydroxyapatite (n-HA).

The internal crystal morphologies can be changed by the addition of different fillers, for instance, nanoparticles, that directly affect the composites’ crystallinity. Figure 1 displays the XRD patterns of pristine polypropylene (PP), PP/20% n-HA/0.3% f-MWCNTs, PP/20% nHA/0.2% f-MWCNTs, PP/20% nHA/0.1% f-MWCNTs and n-HA (JCPD: 09-0432). The maximum crystalline peak observed from XRD patterns at around 2θ = 14.1 for each specimen, except for nHA specimen Pristine PP is categorized as ortho-rhombic symmetry. In this symmetry, all axes have different lengths and are assigned as a, b, and c, i.e. 6.6, 20.8, and 6.5 ^o^A^27^. Besides, the angle between the axes is assigned as α, γ, and β and all equal to 90°. According to Hullerman and co-associates, PP has a degree of crystallinity of 35%^28^. Based on the literature, pristine PP display pattern characteristics at 2θ as 14.1, 16.9, 18.5, 21.1 and 21.7°, corresponded to 110, 040, 130, 111 and 041 planes of α- PP crystals, whereas, the characteristics peaks of n-HA allocated at 26.0°, 28.2°, 28.9°, 31.89° and 34.1°, interrelated to 002, 102, 210, 211 and 301^29^. The XRD pattern of examined specimen i.e. PP/20% n-HA/0.3% f-MWCNTs, PP/20% n-HA/0.2% f-MWCNTs and PP/20% n-HA/0.1% f-MWCNTs also displayed the identical peak patterns as of pristine PP. Therefore, XRD analysis can conclude that there is no structural change is induced in pristine PP due to the addition of f-MWCNTs and n-HA at different compositions.

### Scanning electron microscope (SEM)

**Figure 2:**
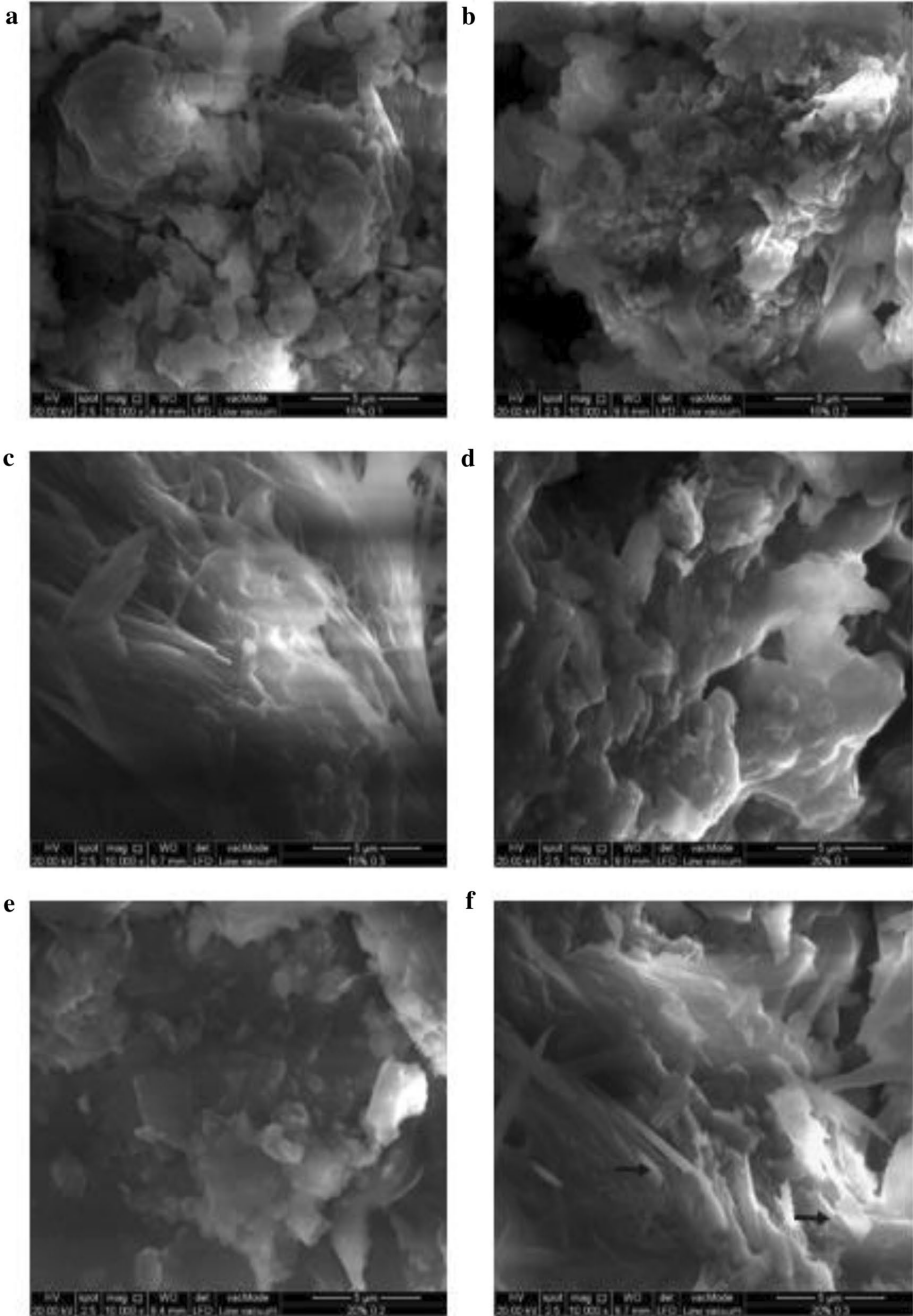
Specimen’s SEM morphologies (**a**) PP/15% n-HA/0.1% f-MWCNTs, (**b**) PP/15% n-HA/0.2% f-MWCNTs, (**c**) PP/15% n-HA/0.3% f-MWCNTs, (**d**) PP/20% n-HA/0.1% f-MWCNTs, (**e**) PP/20% n-HA/0.2% f-MWCNTs, (**f**) PP/20% n-HA/0.3% f-MWCNTs.

SEM image of each specimen are depicted in Fig. 2. Furthermore, it was also observed that dimensions of the fillers of the composite measured in nanometer, i.e. 5 μm. Generally, f-MWCNTs display good dispersion in the polymeric matrix of these composites as highlighted by black arrows in Fig. 2f. In specimen of PP/20% n-HA/0.1%f-MWCNTs (Fig. 2d) and PP/20% n-HA/0.3% f-MWCNTs (Fig. 2f) experience fine cluster due to nHA agglomeration, moreover, these specimens contain higher content of n-HA. Prevention of nHA clustering is difficult, particularly in high loading filler due to their significant surface areas. This is a typical characteristic of polymer nanocomposites incorporated with higher filler contents. In the previous studies, the structure of PP/n-HA nanocomposites has already been reported. The same clustering behavior of n-HA is observed in the polymer matrix of PP/n-HA with high filler fillings^11^. Moreover, Kai et al.^30^ researched on PP/n-HA/hexagonal boron nitride (hBN) nanocomposite, and the results showed that the n-HA homogenous dispersion could be attained at its low content in PP matrix as a large amount tends to aggregate. Thus, the low content of n-HA in PP nanocomposites are restricted from biomedical related fields such as implantation because of poor biocompatibility. To attain PP composites’ better biocompatibility and bioactivity, the minimum content of n-HA is needed, i.e. at least 20%^30^. Another study conducted by Esperanza et al. using polycaprolactone (PCL)/n-HA nanocomposite also demonstrated that high loading of n-HA offers fibrous appearance, while low loading display porous appearance in the PCL/n-HA^31^. Thus for industrial purposes, a low amount of filler is recommended to prevent nanomaterials agglomeration^32^. However, polymer nanocomposites for applications related to bone replacement require a higher amount of n-HA. It is to facilitate adhesion and osteoblasts growth on polymer nanocomposites^33^.

### Thermogravimetric analysis (TGA)

**Figure 3:**
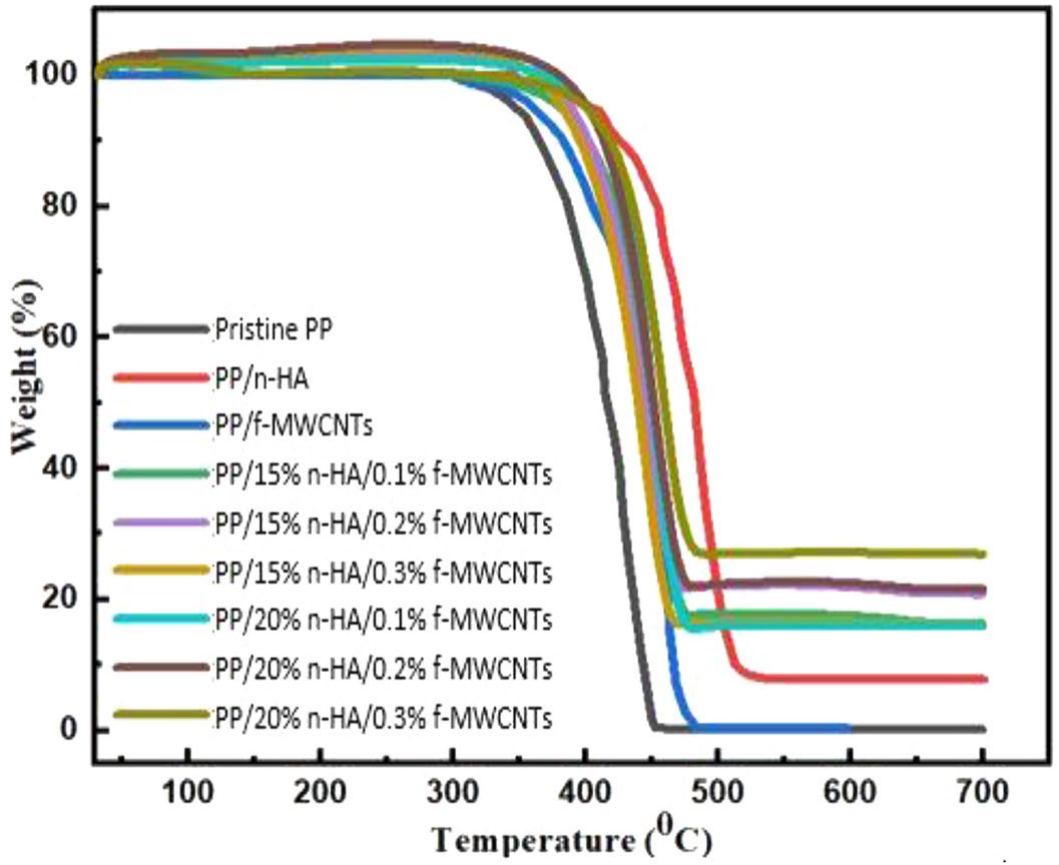
Thermogravimetric analyzes curve for nanocomposites.

TGA studies were carried out on a prepared specimen; the difference of specimen nanocomposites weight was determined as temperature function, from 50 to 700 °C. Figure 3 illustrates the TGA curve of nanocomposites specimen. The weight loss of each specimen differs, for instance, PP/20% n-HA/0.3% f-MWCNTs shows the least weight loss of 70% compared to PP/20% n-HA/0.1% f-MWCNTs displayed the maximum weight loss on TGA studies, i.e. 82%. The slight weight loss of PP/20% n-HA/0.3% f-MWCNTs and PP/20% n-HA/0.2% f-MWCNTs with rising temperature from 100 to 130 °C noted. It was caused due to the water evaporation from these specimens^34^. The TGA curves of the second weight loss of the specimen were observed from 400 to 495 °C. This mass loss of the nanocomposite’s specimen is due to thermal decomposition. At the last stage, the nanocomposites specimen displays a flat profile at the temperature above 495 to 700 °C. It concluded that all the nanocomposites specimen left as a residue after the onset temperatures as the specimen were volatile no longer^35^. The TGA results reflect that the addition of f-MWCNTs and n-HA is beneficial in enhancing the thermal strength of PP^36^.

### Differential scanning calorimetry (DSC)

DSC cooling curve of PP/n-HA/f-MWCNTs helps to determine T_o_ (on-set crystallization temperature); T_c_ (peak crystallization) and ∆H_c_ (crystallization enthalpy) values and listed in Table 1. However, X_c_ (crystallinity degree) of PP/n-HA/f-MWCNTs is calculated using the expression below^37^;

**Table 1:** Specimen investigated thermal and crystallization parameters.

Specimen	T_o_	T_c_	$\Delta \text{Hc}$(J/g)	X_c_	HDT(^o^C)	T_5%_
PP/15% n-HA/0.1% f-MWCNTs	126.8 ± 0.3	123.0 ± 0.2	85.5	48.2	133.3 ± 0.8	447 ± 2.1
PP/20% n-HA/0.1% f-MWCNTs	126.9 ± 0.1	123.4 ± 0.1	84.6	50.7	134.9 ± 0.5	448 ± 1.3
PP/15% n-HA/0.2% f-MWCNTs	126.7 ± 0.1	123.2 ± 0.1	85.2	48.7	134.4 ± 0.5	446 ± 1.3
PP/20% nHA/0.2% f-MWCNTs	126.3 ± 0.1	123.1 ± 0.2	85.8	48.8	134.1 ± 0.4	447 ± 1.4
PP/15% n-HA/0.3% f-MWCNTs	126.2 ± 0.1	123.1 ± 0.1	87.3	49.3	133.5 ± 0.3	449 ± 1.4
PP/20% n-HA/0.3% f-MWCNTs	127.7 ± 0.2	123.5 ± 0.3	81.7	49.1	135.5 ± 1.2	452 ± 2.5

$$Xc \left(\%\right)= \frac{100 \Delta \text{Hc }}{\Delta \text{Hm }(1-\upphi )}$$

∆H_m_ = Melting enthalpy of crystalline PP (100%), i.e. 209 J/g. $\upphi$ = Weight fraction of the nanocomposite’s filler. ∆H_c_ = Crystallization enthalpy, J/g. X_c_ = Crystallinity degree, %.

**Figure 4:**
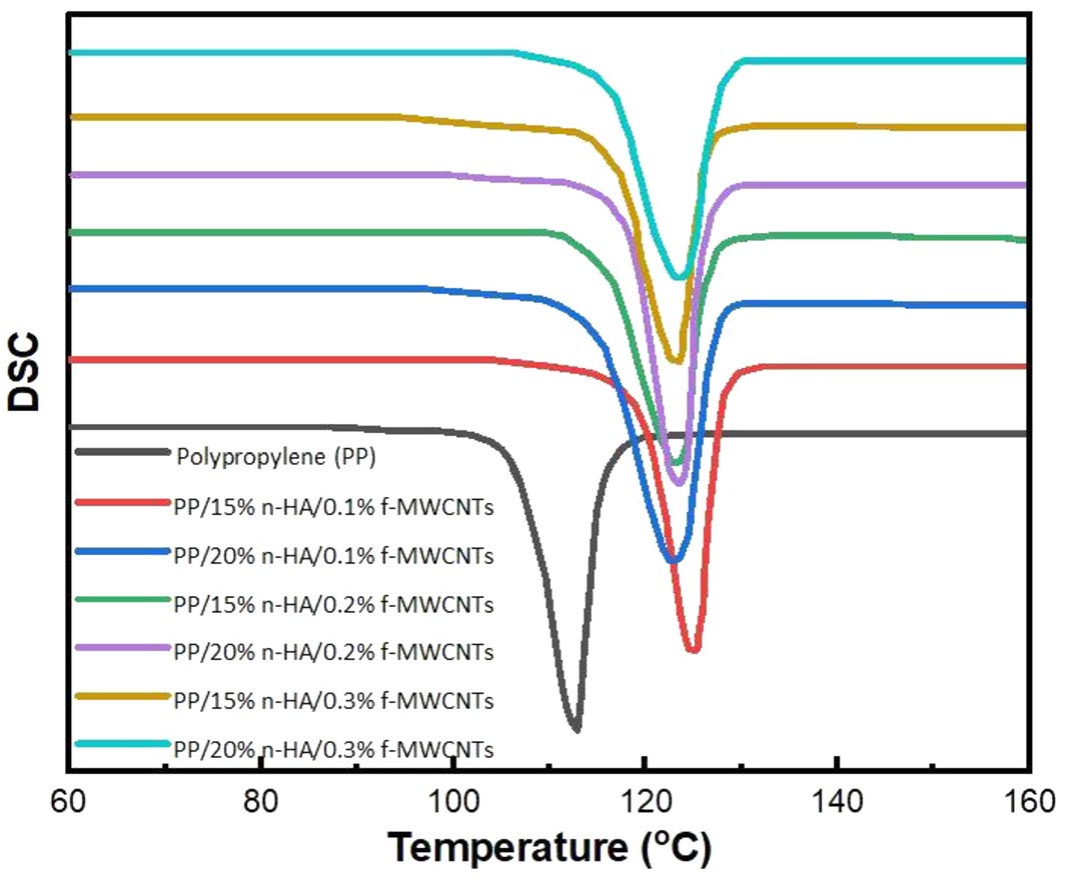
Differential scanning calorimetry curve of polypropylene and nanocomposites.

It is visible through Fig. 4 and Table 1 that on-set crystallization temperature, as well as peak crystallization, gradually inclines as the content of n-HA increases. The T_c_ value of un-contaminated PP is 115.69 °C, and inclines to 121.4 and 123.9 °C by the addition of 15% and 20% n-HA, respectively^11^. With the addition of n-HA content in the composite to 15%, T_c_ increases to 123.0 and 123.1 °C by incorporating with 0.1 and 0.3% f-MWCNTs, respectively. Furthermore, there is no raise in the T_c_ of PP/20% n-HA composite by the addition of f-MWCNTs. It shows that both f-MWCNTs and n-HA for PP crystallites behave as nucleating agents^38^. Nevertheless, f-MWCNTs effectiveness for nucleating PP is mainly based on the content of n-HA. Henceforth, n-HA and f-MWCNTs have competition with each other for the PP macromolecules nucleation. With 15% of n-HA content, f-MWCNTs are considered to be more beneficial for nucleating PP in the composite. Thus, f-MWCNTs show no effect with higher content of n-HA, particularly due to the nHA fillers serious agglomeration. The X_c_ values drop slightly with the increase in the n-HA content. With further increase in the content of f-MWCNTs i.e. 0.1 to 0.3%, improve its crystallinity. Also, PP melting temperature inclines by 2–4.3 °C by combining 15 and 20% n-HA and 0.1 and 0.3% f-MWCNTs respectively^33,36^.

### Young’s modulus and tensile strength

**Figure 5:**
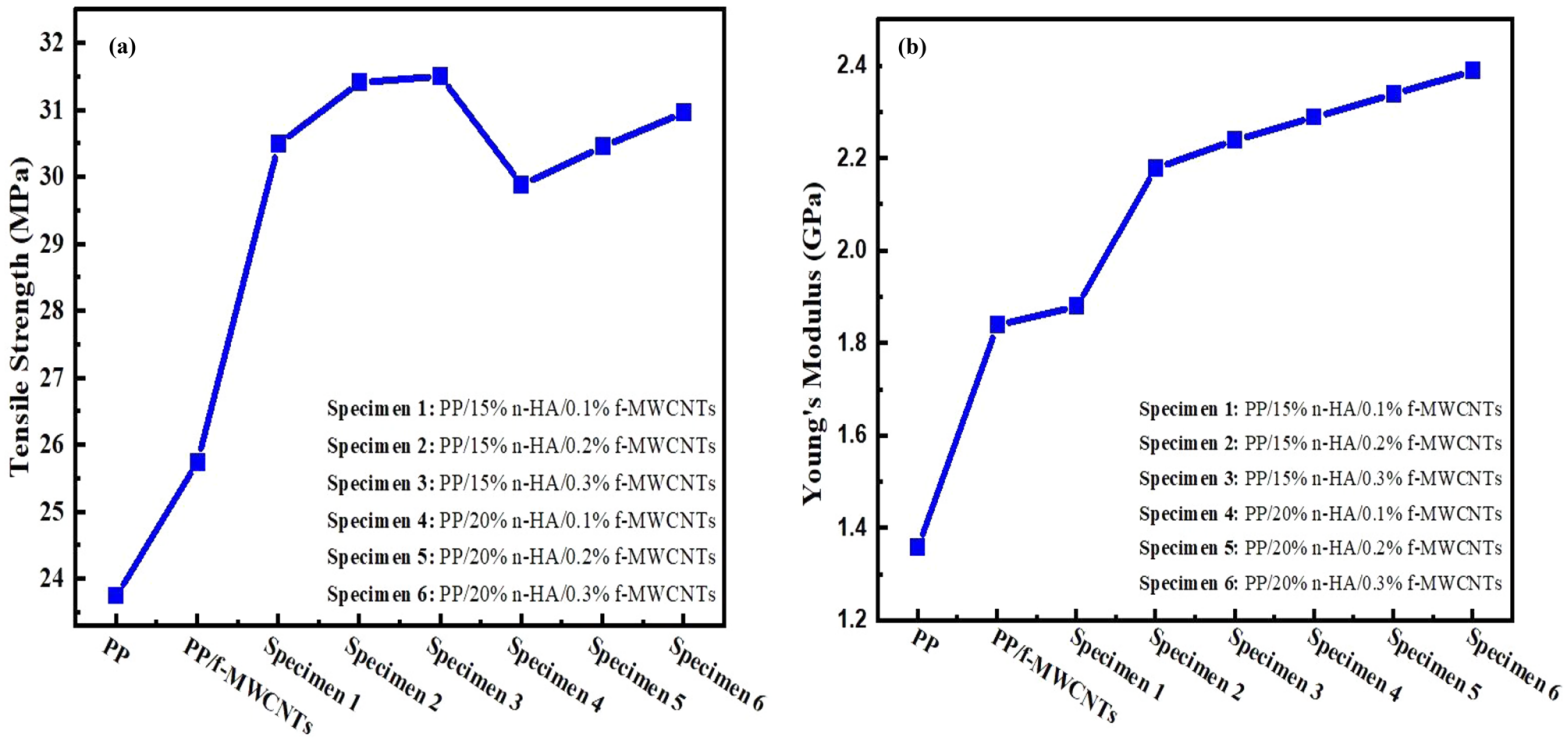
(**a**) Variations of tensile strength with n-HA for nanocomposites. (**b**) Variations of Young’s modulus with nHA for nanocomposites.

Young’s modulus and tensile strength as a function of nHA of PP/n-HA/f-MCWNTs composite depicted in Fig. 5(a–b). The tensile strength is considerably improved by the addition of f-MCWNTs in PP/n-HA composite due to significant aspect ratio as well as unique mechanical strength^39^. As stated earlier, significant content of n-HA added to the polymer bio-composites considered for bone implants that have the capability of inducing osteoblasts growth as well as adhesion. In the past, Liu and Wang prepared composite PP/HA protected with 10, 20, and 25 vol. % micro-particles of HA that resulted in a decrease in polypropylene tensile strength from 29.55 MPa to 26.32 MPa, 22.34 MPa, and 20.16 MPa respectively by the addition of 10, 20, and 25 vol. % of HA^39^. Moreover, this work used 15 and 20% wt.% of n-HA, corresponding to 4.80, 6.67 vol. % respectively. However, PP/n-HA volume contents are extremely lower compared to PP/n-HA micro-composites prepared by Liu and Wang^17^. Figure 5(a) revealed that polypropylene tensile strength starting from 29 MPa, and inclined 31 MPa by the addition of n-HA, i.e. 15% (4.80 vol. %), but it reduced to 30.2 MPa by the addition of 20% (6.67 vol. %) n-HA content. Based on the tensile strength results, the addition of n-HA content lower than 20% can enhance the tensile strength of PP. Nevertheless, polypropylene tensile strength can further be enhanced via hybridizing n-HA with f-MWCNTs^39^. Based on Fig. 5(b) it shows the tensile test of this study discloses that Young’s modulus and tensile strength of PP/n-HA increase particularly with the addition of 0.2 wt.% of f-MWCNTs. However, with the addition of f-MWCNTs of more than 0.2 wt.%, there is only minimal improvement on both Young’s modulus and tensile strength. Likewise, single-walled carbon nanotubes (SWCNTs) were also chosen to strengthen nanocomposites such as n-HA/chitosan^40^. As oppose to SWCNTs/n-HA/chitosan, and MWCNTs/n-HA/PMMA (Polymethyl methacrylate) is not recommended for orthopedics application^39^. Table 2 illustrates the comparison of tensile and Young’s modulus of different nanocomposites.

**Figure 6:**
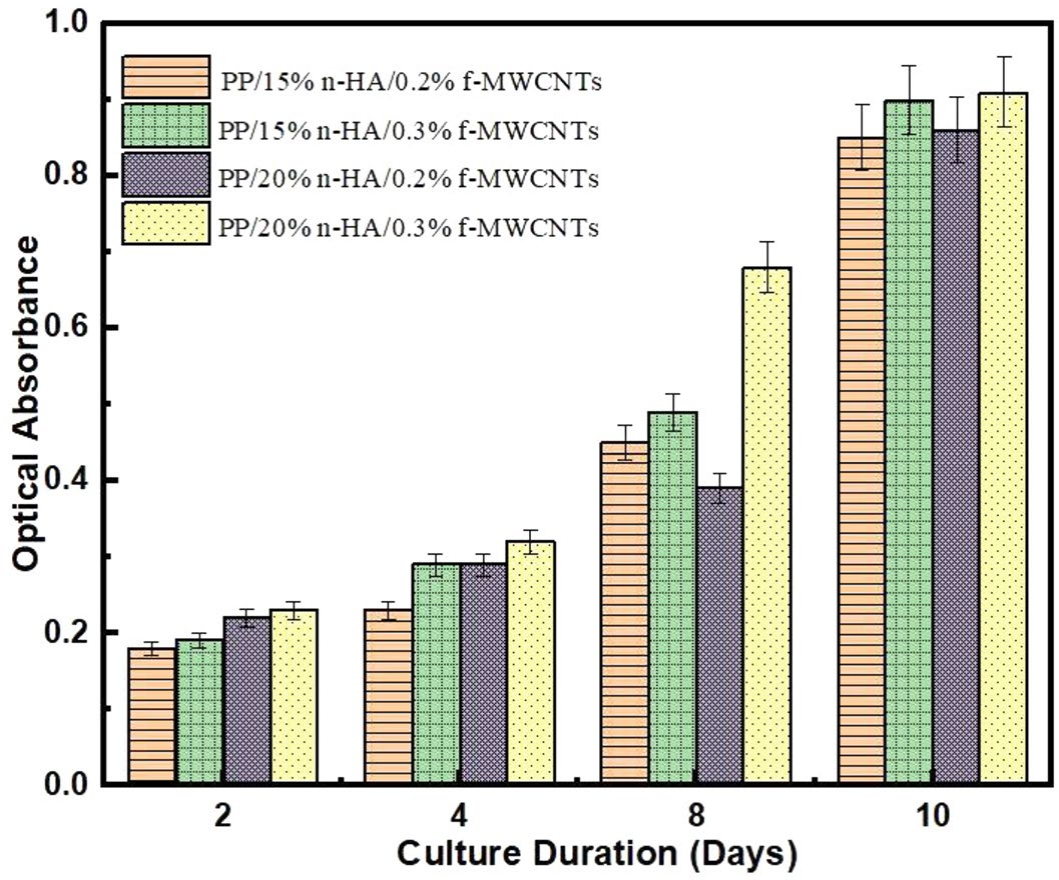
MTT assay analysis.

**Table 2:** Comparison of tensile and Young’s modulus of different nanocomposites.

Specimen	Nanotubes composition (wt. %)	Tensile strength (MPa)	Young modulus (MPa)	References
Poly (lactide-co- glycol)/multi-walled carbon nanotubes	3	35	510.9	^21^
Polypropylene fermarate/single walled carbon nanotubes	0.02	51.5	1300	^45^
Chitosan/multi-walled carbon nanotubes	5	47.2	79.2	^46^
PP/n-HA/f-MWNTs	0.2	31.5	2200	Present study

### MTT cell proliferation assay

Generally, HA nanomaterials improve the new bone tissue growth by increasing osteointegration and osteoblast adhesion on their exterior^41^. The PP/f-MWCNTs/n-HA osteoblast proliferation rate is expressed using MTT assay, Fig. 6. Based on the MTT graph, the optical absorbance indicates the osteoblast growth (multiplication of osteoblast) rate concerning the culturing period (days). The higher value of absorbance reflects an inclination in the rate of reproduction of osteoblast, whereas, lower value describes a reduction in the rate of reproduction of osteoblast^42^. Figure 6 shows that the osteoblastic proliferation gradually increases on each specimen as the culturing period increased up to 10 days. In comparison with all specimens, PP/20% n-HA/0.3% f-MWCNTs-composite reveals a higher osteoblast growth rate, 8 days. It reflects that PP biocompatibility is significantly enhanced by the addition of f-MWCNTs and n-HA at different compositions. Based on the literature, CNTs possibly generate cytotoxicity upon inhalation and disclosure to human cells^43^. Moreover, few researchers have found low viability in epidermal keratinocytes. On the other hand, some researchers have stated a satisfactory cellular connection with nanotubes by sowing with osteoblast and neurons^44^. In one of the studies, PP/20% n-HA specimen was prepared using extrusion, and injection molding technique for human bone replacement. The specimen MTT test showed an increase of only 19% cell viability after 7 days which is comparatively lower than the present studies^30^. Therefore, the present study confirms that the addition of f-MWCNTs into the PP/n-HA polymer matrix displays no negative influence on the viability and osteoblast proliferation but a relative increase of these properties.

Lastly, the f-MWCNTs with different weights were loaded to the nanocomposites targeting to study f-MWCNTs additions effect on their properties and biocompatibility. Therefore, 0.2 and 0.3 wt.% f-MWCNTs are added to PP/n-HA suitable for applications related to biomedical fields. Essentially, experimental measurements only testify that the nanotubes additions affect the PP/f-MWCNTs/n-HA performance. The results from the experiment indicate that for 0.2 wt.% f-MWCNTs with PP/n-HA have a less or more similar effect on the nanocomposites than with 0.1 and 0.3 wt.%. This concludes that PP/n-HA can be significantly improved by adding 0.2 wt.% f-MWCNTs.

## Conclusion

In conclusion, this research presented the design and testing of hybrid composite for biomedical applications, especially for specific human bone part replacements, for instance, subchondral bone. Herein, this study demonstrates a melt compounding approach to fabricate a nanocomposite of f-MWCNTs, and n-HA reinforced PP for future generation biomedical applications. Through this approach, it is possible to enhance material homogeneity, moreover, optimize the amount of f-MWCNTs and n-HA in the PP matrix. The nanocomposites were successfully fabricated via a melt processing approach. Hybrid nanocomposites inborn the characteristics of individual fillers through developing materials with appropriate mechanical and biocompatibility properties. The analysis showed that Young’s modulus of PP composites inclines with the low filling of f-MWCNTs and n-HA, i.e. 0.1, 0.2, 0.3 wt.%, and 15–20 wt.%, respectively. An increase in f-MWCNTs more than 0.2 wt.% decreases the tensile ductility of PP composites. Finally, the MTT assay study has revealed the feasibility and proliferation activity of osteoblasts cultured on PP/n-HA/f-MWCNTs mixes. Moreover, PP/20% n-HA/0.2% f-MWCNTs nanocomposite is more biocompatible compared to 0.1% and 0.3% f-MWCNTs filling. Based on the results attained through characterization studies, the prepared nanocomposites, i.e. PP/20% n-HA/0.2% f-MWCNTs are the more suitable sample for light-loaded bone implants such as the subchondral bone i.e. patella (Young’s modulus: 1.6 ± 0.7 GPa), and cartilage (Young’s modulus: 0.5 to 0.9 MPa). In the future, further studies are needed to better assess the change of nanocomposite structure, particularly at higher filler contents of f-MWCNTs.

## Experimental

### Materials

Functionalized multi-walled carbon nanotubes (f-MWCNTs) (21.36 wt% oxygen content, pH-7) were obtained from the previous studies using sonication treatment method^47^. Polypropylene (PP) pellets (average M_w_ 14,000 ~ 3700) and Dodecyl-amine were obtained from MERCK, Malaysia. Ethanol (C_2_H_5_OH) (99%), Sodium hydroxide (NaOH), phosphoric acid (HNO_3_) (85%), and calcium chloride (CaCl_2_) were purchased from Systherm Chemicals, Malaysia.

### Preparation of hydroxyapatite nanorods (n-HA)

A mixture of CaCl_2_, HNO_3_, dodecyl-amine (Templating agent), and de-ionized water was prepared in a beaker with a molar ratio of 2:2:1:60. NaOH (sodium hydroxide) (6 mol/liter) was added to the mixture until pH 10 was obtained. The pH-maintained mixture stirred for 30 min using a magnetic stirrer to obtain a milky suspension. The milky suspension then placed in an autoclave for 12 hr, while maintaining the oven temperature at 100 °C. The precipitate obtained after 12 hr, later washed several times with de-ionized water. The washed precipitate was placed in a beaker that already contains an equal volume of de-ionized water and ethanol. The mixture is stirred for 8 hr and the mixture was frozen and dried, to obtain the desired product in powder form.

### Preparation of PP/n-HA/f-MWCNTs

The PP/n-HA/f-MWCNTs compositions were weighted, as stated in Table 3. Each specimen is heated for 9 min on a hot plate with three-time intervals, i.e. 3 min at 215 °C, 3 min at 230 °C, and 3 min at 180 °C. The heated specimen was transferred to the oven for 24 hr at 70 °C. After 24 hr, the dried specimen is crushed using a crusher to obtain the final product.

**Table 3:** Specimen composition.

No	Specimen	PP (wt. %)	n-HA (wt. %)	f-MWCNTs (wt. %)
1	PP/15% n-HA/0.1%f-MWCNTs	84.9	15	0.1
2	PP/15% n-HA/0.2%f-MWCNTs	84.8	15	0.2
3	PP/15% n-HA/0.3%f-MWCNTs	84.7	15	0.3
4	PP/20% n-HA/0.1%f-MWCNTs	79.9	20	0.1
5	PP/20% n-HA/0.2%f-MWCNTs	79.8	20	0.2
6	PP/20% n-HA/0.3%f-MWCNTs	79.7	20	0.3

## Characterization analysis

### Structure and morphology

A diffractometer instrument (Siemens, D500) with Cu-Kα radiation (λ = 0.154 nm), maintained at 40 kV and 30 mA, was used to examine the X-ray diffraction patterns of each specimen. However, the data was read at 10 °C to 40 °C with a 0.02°/s scanning rate. FE-SEM (Field emission scanning electron microscope) was used to observe the composites fracture surface morphology. Before scanning the electron microscope, the specimen surface was covered with a carbon thin layer.

### Mechanical strength

The prepared specimen’s mold used to conduct tensile testing is based on Type 1 tensile bar dimension (50 mm gauge-length) and thickness (6 mm) as stated by ASTM (D638-08) international, which was in dumbbell shape mold. The tensile testing was carried out using an Instron tester (M-5567), with a cross-head speed of 10 mm/min. Whereas, Young’s modulus testing was conducted using an extensometer. A minimum of three specimens for each composition was examined, and average values are taken for further analysis.

### Thermal study

TA (Thermal analyzer instrument, model 2910), used for DSC (differential scanning calorimetry) under protective N_2_ atmosphere. The thermal history of each specimen is removed by-pass through the heating cycle process that starts at 100 °C/min from ambient to 200 °C for 3 min. After the heating cycle, each specimen cooled at 10 °C/min, and cooling traces were noted. Another heating and cooling cycle was conducted at the same rate and noted the heating and cooling curves.

Another thermal analyzer instrument, TGA-50, was used with the same atmosphere condition as DSC but at a heating rate of 20 °C/min. The change in weight percentage was recorded for each specimen from 50 to 700 °C. Furthermore, degradation temperature at 5% weight loss (T_5%_) can be calculated accordingly.

### MTT (dimethyl thiazolyl diphenyl tetrazolium) cell proliferation assay

MTT assay was conducted to observe the nanocomposites’ cell viability. The specimen were seeded with 100 μl cell suspension (10^4^ cells) with dimension (4 × 4x0.5 mm) sited in a 96-well plate and incubated at 37 °C in environment conditions (5% carbon dioxide/95% air) for 2,4, 8, and 10 days, correspondingly. After every 3 days, DMEM, Dulbecco’s Modified Eagle Medium, was replaced. 10 μl MTT solutions were added for 4 h after incubation. The cells metabolized MTT, formazan product yielding inside at this situation. To dissolve formazan crystal, 100 μl of SDS (10%) in 0.01 M HCl was added. The multimode detector was used to measure the resulting solution’s light absorbance with 640 nm wavelength as reference. For comparison, negative control (only DMEM) and positive control (osteoblast in 96-well plate with similar seeding density as the specimen) were also conducted. All assays were carried out in triplicate, and statistical differences (p < 0.05) were determined via the Wilcoxon signed-rank test.
